# Lipidomic insights into the response of *Arabidopsis* sepals to mild heat stress

**DOI:** 10.1007/s42994-023-00103-x

**Published:** 2023-06-06

**Authors:** Zican Chen, Weronika Jasinska, Muhammad Ashraf, Leah Rosental, Jung Hong, Dabing Zhang, Yariv Brotman, Jianxin Shi

**Affiliations:** 1 Joint International Research Laboratory of Metabolic and Developmental Sciences, State Key Laboratory of Hybrid Rice, School of Life Sciences and Biotechnology, Shanghai Jiao Tong University, Shanghai, 200240 China; 2 Department of Life Sciences, Ben Gurion University of the Negev, Beersheva, 84105 Israel; 3 School of Agriculture, Food and Wine, University of Adelaide, Urrbrae, SA 5064 Australia; 4 Yazhou Bay Institute of Deepsea Sci-Tech, Shanghai Jiao Tong University, Shanghai, 200240 China

**Keywords:** Floral development, Heat shock protein, Lipidomic profiling, Stress response, Voltage-dependent anion channel protein

## Abstract

Arabidopsis sepals coordinate flower opening in the morning as ambient temperature rises; however, the underlying molecular mechanisms are poorly understood. Mutation of one heat shock protein encoding gene, *HSP70-16*, impaired sepal heat stress responses (HSR), disrupting lipid metabolism, especially sepal cuticular lipids, leading to abnormal flower opening. To further explore, to what extent, lipids play roles in this process, in this study, we compared lipidomic changes in sepals of *hsp70-16* and *vdac3* (mutant of a voltage-dependent anion channel, VDAC3, an HSP70-16 interactor) grown under both normal (22 °C) and mild heat stress (27 °C, mild HS) temperatures. Under normal temperature, neither *hsp70-16* nor *vdac3* sepals showed significant changes in total lipids; however, *vdac3* but not *hsp70-16* sepals exhibited significant reductions in the ratios of all detected 11 lipid classes, except the monogalactosyldiacylglycerols (MGDGs). Under mild HS temperature, *hsp70-16* but not *vdac3* sepals showed dramatic reduction in total lipids. In addition, *vdac3* sepals exhibited a significant accumulation of plastidic lipids, especially sulfoquinovosyldiacylglycerols (SQDGs) and phosphatidylglycerols (PGs), whereas *hsp70-16* sepals had a significant accumulation of triacylglycerols (TAGs) and simultaneous dramatic reductions in SQDGs and phospholipids (PLs), such as phosphatidylcholines (PCs), phosphatidylethanolamines (PEs), and phosphatidylserines (PSs). These findings revealed that the impact of mild HS on sepal lipidome is influenced by genetic factors, and further, that *HSP70-16* and *VDAC3* differently affect sepal lipidomic responses to mild HS. Our studies provide a lipidomic insight into functions of HSP and VDAC proteins in the plant’s HSR, in the context of floral development.

**Supplementary Information:**

The online version contains supplementary material available at 10.1007/s42994-023-00103-x.

## Introduction

Heat stress (HS) poses severe adverse effects on plant growth and development; even a slight increase in temperature can cause significant losses in crop yield (Mittler et al. 2012). It is well established that plant reproductive organs are more sensitive than vegetative organs to HS (He et al. 2019; Zhang et al. 2017); thus, HS affects more severely plant reproductive development (Rutley et al. 2022; Wang et al. 2021). To secure food supply, it necessitates establishing a comprehensive understanding of the molecular mechanisms governing the plant defenses against HS.

During their life cycle, plants can experience mainly two forms of HS: mild HS with moderately elevated temperatures (about 5 °C over the optimal temperature) and severe HS with extremely increased temperatures (about 20 °C or more than the optimal temperature); the later has been mostly studied (Groot et al. 2017; Jagadish et al. 2021). Both stresses differently affect plant growth and development, and plants respond to them differently, at both physiological (Groot et al. 2017; Sato et al. 2000) and molecular (Haider et al. 2021; Mittler et al. 2012; Ran et al. 2020) levels. On the one hand, HS can damage thermos-labile macromolecules, such as proteins and membranes and, thus, their functions; alternatively, plants can sense rising temperatures, and activate a series of changes to establish effective defense system for plant survival and reproduction, under HS conditions (Guihur et al. 2022; Haider et al. 2021; Jagadish et al. 2021; Mittler et al. 2012). Therefore, deleterious effects of HS, either reversible or irreversible, are complex, depending on many factors, such as the rate of rising temperature, the intensity of the risen temperature, its duration, and the presence of other stresses.

The conserved HS responses (HSR) in plants are typically composed of several steps. During thermal perception and signaling, plants sense a mild temperature rise, likely as an HS warning, through uncharacterized cellular thermos-sensors, and translate it into an HSR signal; during HSR, plants quickly synthesize superfluous heat-induced proteins, particularly heat shock proteins (HSPs), to repair damaged macromolecules, transduce HS signals, and to produce thermo- and reactive oxygen species (ROS)-protective metabolites; and during survival, plants accumulate many protective metabolites, including amino acids (such as proline) and sugars (for instance trehalose) to protect native proteins and membranes, and heat-induced molecular chaperones to repair heat-aggregated proteins (Guihur et al. 2022). It is also well established that pre-exposing plants to a non-lethal high-temperature, the so-called ‘priming’, induces acquired thermotolerance, and increases plant survival and performance under a subsequent HS (Guihur et al. 2022; Jagadish et al. 2021; Mittler et al. 2012).

It is generally accepted that 27 °C is not a harmful temperature for most plants, including *Arabidopsis* (Groot et al. 2017; Mittler et al. 2012; Sato et al. 2000), as a 27 °C treatment does not trigger expression of many HSR markers (Kumar and Wigge 2010; Mittler et al. 2012) and the transcriptomic response of *Arabidopsis* to 27 °C differs significantly from that of a simple HS treatment heating from 22 to 37 °C (Mittler et al. 2012). Notably, different from HSR priming that leads to a rather short-term acclimation and acquired thermotolerance, a 27 °C treatment usually results in longer term adaptation and developmental reprogramming (Kumar and Wigge 2010), thereby indicating different thermos-signaling and responses in these two HSRs. In addition, *Arabidopsis* can partially adapt to mild HS, over generations (Groot et al. 2017). Therefore, elucidation of the plant acquired thermotolerance, in response to a mild prior warming, is essential to lay a fundamental base for breeding new thermos-resistant crops (Guihur et al. 2022; Jagadish et al. 2021; Krishna 2003).

In plants, heat sensing occurs at the plasma membrane (PM), and it is HS-induced changes in the membrane fluidity that activates a PM calcium channel and the subsequent HSR (Guihur et al. 2022; Mittler et al. 2012; Saidi et al. 2009). As membrane fluidity is determined by the ratio of saturated-to-unsaturated lipids, and all plants adjust membrane fluidity in response to temperature changes by altering lipid saturation and composition (Guihur et al. 2022; Saidi et al. 2010), lipid metabolism is essential for thermos-sensing and Ca^2+^-dependent HSR. In addition, changes in PM membrane fluidity might trigger lipid signaling; these lipid signals include inositol-1,4,5trisphosphate (IP3) that is generated from phosphatidylinositol 4,5-bisphosphate (PIP2), by phosphoinositide (PI)-specific phospholipases (PLCs) (Gao et al. 2014; Ren et al. 2017), phosphatidic acid (PA) (Mittler et al. 2012), and polyunsaturated fatty acids (PUFAs) (Moreno et al. 2012; Mueller et al. 2017). Furthermore, PM fluidity alterations also impact channel activity of other ions in addition to Ca^2+^, in which various lipid molecules are involved, such as PUFAs with Kv channel (Moreno et al. 2012), phytosterols with voltage-dependent anion channels (VDAC) (Mlayeh et al. 2010, 2017; Saidani et al. 2021), and PI with K^+^ channels (Liu et al. 2005). Nevertheless, although membrane fluidity is, to a great extent, determined by FA desaturase and lipases (Upchurch 2008), to date, the explicit roles of lipids in plant thermos-sensing and transduction remain to be elucidated.

Sepals, the outmost organ of a mature *Arabidopsis* flower, rich in various chemicals, protect developing reproductive organs inside the bud from both biotic and abiotic stresses (Chen et al. 2019; Roeder 2010). As *Arabidopsis* sepals are closely associated with flower opening, in the morning, as ambient temperature rises (van Doorn and Van Meeteren 2003), and a mutation in the heat shock protein encoding gene (*HSP70-16*) impairs genetic HSR responses, disrupts lipid metabolism, and causes defective flower opening (Chen et al. 2019; Ran et al. 2020), it is intriguing to know, to what extent, lipids, particularly, glycerolipids, play roles in the sepal thermos-sensing and HSR, in which ion channel activities are involved (Balogh et al. 2013; Upchurch 2008).

**Fig. 1:**
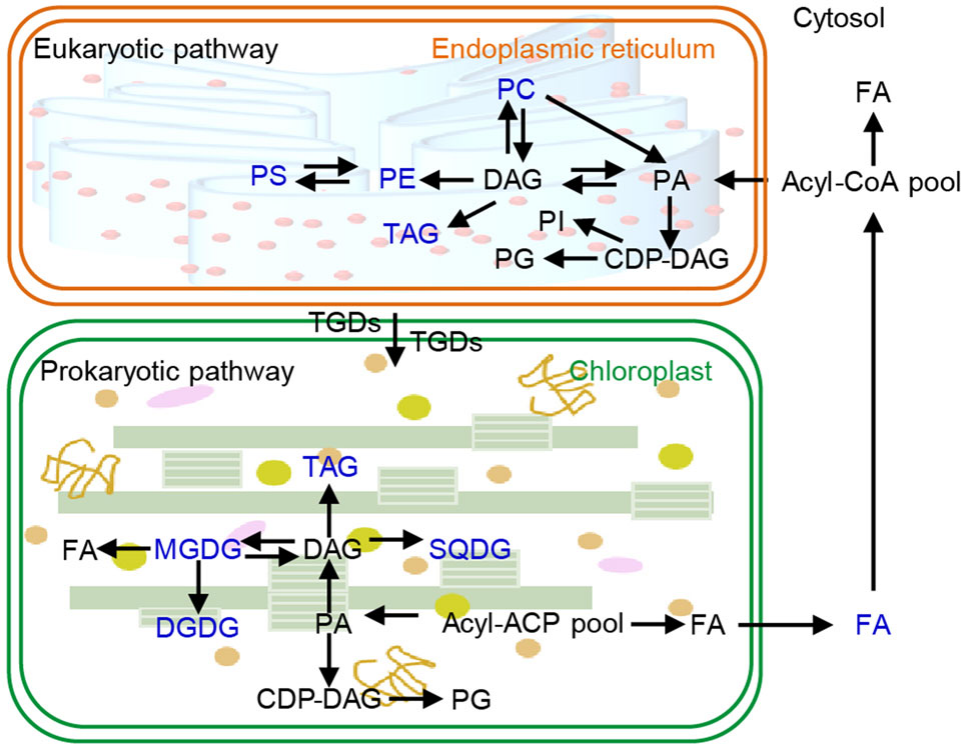
General overview of the de novo fatty acid synthesis and glycerolipid synthesis under severe heat stress in *Arabidopsis* leaves. Severe heat stress turns the chloroplastic glycerolipid synthesis from the prokaryotic pathway to the eukaryotic pathway. *DAG* diacylglycerol, *DGDG* digalactosyldiacylglycerol, *FA* fatty acid, *MGDG* monogalactosyldiacylglycerol, *PC* phosphatidylcholine, *PE* phosphatidylethanolamine, *PG* phosphatidylglycerol, *PI* phosphatidylinositol, *PS* phosphatidylserine, *SQDG* sulfoquinovosyldiacylglycerol, *TAG* triacylglycerol, *TGD* trigalactosyldiacylglycerol proteins. FA connects the two pathways, and TGDs sitting at the chloroplastic outer and inner envelope membranes form a transporter complex mediating ER-to-plastid lipid trafficking. Blue-colored lipid molecules are those significantly altered in sepals of *Arabidopsis* with different genetic backgrounds

Glycerolipids, the main component of plant membranes, play important roles in floral development and stress response (Devaiah et al. 2006; Horn and Benning 2016). Although glycerolipids are known to be produced from both plastidic (prokaryotic) and extraplastidic (eukaryotic) glycerolipid pathways, and fatty acids (FAs) are integrated into glycerolipids, via the same two pathways in *Arabidopsis* leaves (Fig. 1), information on glycerolipid biosynthesis in *Arabidopsis* sepals remains unknown. Currently, knowledge about HS on lipidomic profiles is derived predominantly from studies in *Arabidopsis* leaves. Here, severe HT stress reduces the unsaturation of glycerolipids and activates eukaryotic pathways for chloroplastic galactolipid synthesis, whereas it inactivates prokaryotic glycerolipid synthesis in *Arabidopsis* leaves (Higashi and Saito 2019). However, it is also reported that severe HT stress, alone, only reduces the unsaturation of one glycerolipid species, phosphatidylglycerol (PG) (Burgos et al. 2011).

Information regarding mild HS is limited; the only study (Qin et al. 2020) reported that mild HS (30 °C) inactivates the prokaryotic pathway, but slightly activates the eukaryotic pathway in *Arabidopsis* leaves. Considering the significant difference in glycerolipid profiles between leaves and flowers in *Arabidopsis* (Devaiah et al. 2006), it is interesting to investigate whether and how glycerolipids in *Arabidopsis* sepals respond to mild HS (27 °C).

In this study, using a lipidomic approach, we compared lipidomic changes in sepals of *hsp70-16* and *vdac3*, the latter being a mutant of the interactor of HSP70-16, VADC3 (Ashraf et al. 2021), grown under normal (22 °C) and mild HS (27 °C) temperatures. Our results revealed that *HSP70-16* and *VDAC3* differently affect lipidomic responses of sepal to mild HS, providing novel and lipidomic insights into the functions of HSP and VDAC proteins in the HSR in plants.

## Results

### Lipidomic profiling in Arabidopsis sepals at stage 12

Our previous studies showed that mild HS can severely affect lipid metabolism, particularly cuticular lipid metabolism, and related gene expression in sepals and that it is intensified by the mutation of *HSP70-16* (Chen et al. 2019; Ran et al. 2020). To explore to what extent the mild HS affects sepal lipids, we used ultra-performance liquid chromatography–Fourier transform–mass spectrometry (UPLC–FT–MS) and performed a lipidomic profiling in sepals, at stage 12, grown under both normal (22 °C) and mild HS (27 °C) temperatures. UPLC–FT–MS identified in total 239 lipid molecules in all sepal samples, with 140 and 99, respectively, in both positive and negative modes (Table S1).

**Fig. 2:**
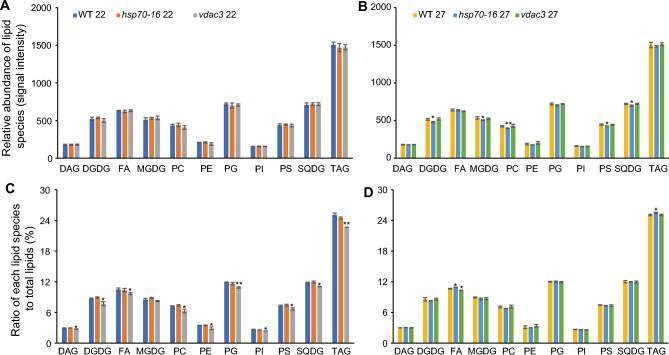
Effects of mild heat stress on lipid classes and their ratios (%) identified in sepals of wild-type (WT), *hsp70-16*, and *vdac3* plants. **A**, **B** Signal intensities of identified 11 lipid classes in sepals of WT, *hsp70-16*, and *vdac3* plants grown under 22 °C and 27 °C, respectively. **C**, **D** Ratio of each identified lipid class to total lipids in sepals of WT, *hsp70-16*, and *vdac3* plants grown under 22 °C and 27 °C, respectively. * and **, significance at *P* < 0.05 and *P* < 0.001, respectively. Refer to legends in Fig. 1 for abbreviation name of each identified lipid class

These identified lipids could be classified into 11 groups (Fig. 2; Table S1), namely, 7 diacylglycerols (DAGs), 18 digalactosyl diacylglycerols (DGDGs), 24 fatty acids (FAs), 18 monogalactosyldiacylglycerols (MGDGs), 15 phosphatidylcholines (PCs), 7 phosphatidylethanolamines (PEs), 27 phosphatidylglycerols (PGs), six phosphatidylinositols (PIs), 16 phosphatidylserines (PSs), 26 sulfoquinovosyldiacylglycerols (SQDGs), and 75 triacylglycerols (TAGs). Among them (Fig. 2C), MGDGs and DGDGs, two galactolipids (GLs) accounted for 8.50% and 8.68% of total lipids, respectively; PCs, PEs, PGs, PIs, and PSs, five phospholipids (PLs), took up 7.22%, 3.46%, 1.92%, 2.61%, and 7.30% of total lipids, respectively; SQDGs, belonging to sulfolipids (SLs) (Horn and Benning 2016; Reszczynska and Hanaka 2020), accounted for 11.80% of total lipids; DAGs and TAGs, each occupied 2.93% and 25.07%, respectively, of total lipids; while FAs, building blocks of all other lipids, seized 10.49% of total lipids. PLs except PGs are extraplastidic lipids synthesized in the ER membrane, and GLs, SLs, and PGs are plastidic lipids biosynthesized exclusively in chloroplasts (Higashi and Saito 2019), whereas TAGs are both plastidic and extraplastidic lipids, mainly synthesized in the ER and also chloroplastic envelop membranes. The diverse lipid molecules identified with UPLC–FT–MS provided the first sepal lipidome in *Arabidopsis* and laid a solid foundation for further comparative study on mild HS-induced lipid remodeling in sepals.

### Effects of *HSP70-16* and *VDAC3* on sepal’s lipidomes are temperature-dependent

**Fig. 3:**
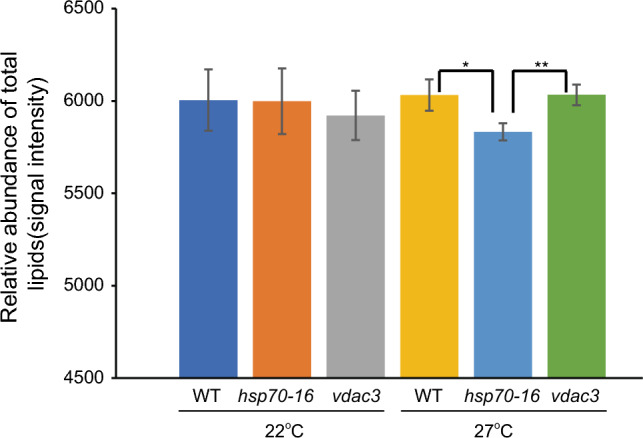
Signal intensities of total lipids identified in sepals of wild-type (WT), *hsp70-16,* and *vdac3* plants grown under both 22 °C and 27 °C. * and **, significance at *P* < 0.05 and *P* < 0.001, respectively

Mutation of *HSP70-16* affects lipid metabolism, particularly cuticle lipid metabolism in sepals, leading to a defective flower opening phenotype at both 22 °C and 27 °C, with a more severe phenotype observed at 27 °C (Chen et al. 2019; Ran et al. 2020). VDAC3 is an interactor of HSP70-16 for seed germination, under 4 °C (Ashraf et al. 2021), and the *vdac3* mutant only showed a similar phenotype as *hsp70-16* at 27 °C (Fig. S1). To examine the changes in sepal total lipids, as affected by both genetics and ambient temperatures, we first compared changes of total lipids in sepals of WT and two mutants, *hsp70-16* and *vdac3*, grown under both 22 °C and 27 °C regimes. Under 22 °C, total lipids in sepals of *hsp70-16* and *vdac3* were not significantly different from those in sepals of WT (Fig. 3; Table S2), suggesting that loss-of-function of *HSP70-16* or *VDAC3* has no significant impact on total lipid metabolism in sepals grown under a normal temperature.

Under 27 °C, levels of total lipids in sepals of *hsp70-16* were significantly lower than those in sepals of either WT or *vdac3*, whereas levels of total lipids, in *vdac3* sepals, were similar to those in sepals of WT (Fig. 3; Table S2). These findings indicated that *HSP70-16* and *VDAC3* have distinct roles in HSR, which differed from their interactive role in seed germination at 4 °C (Ashraf et al. 2021), and confirming again a specific role of *HSP70-16* in the sepal HSR, as established in previous transcriptomic analysis (Ran et al. 2020). The significant reduction of total lipids in *hsp70-16* sepals, grown at 27 °C, was mainly attributed to remarkable reductions in two GLs (DGDGs and MGDGs), two PLs (PCs and PSs), and one SL (SQDGs) (Fig. 2B).

We next investigated changes in ratios of each lipid class, to total lipids, in sepals of WT, *hsp70-16*, and *vdac3* mutants grown under both 22 °C and 27 °C. Under 22 °C, as compared with WT, ratios of each lipid class in sepals of *hsp70-16* were not significantly altered, whereas, except for MGDG, those in sepals of *vdac3* were significantly reduced (Fig. 2C), implying that *VDAC3* is likely to be associated with proper lipid composition, in sepals grown under normal temperature. Under 27 °C, as compared with WT, ratios of FAs and TAGs in sepals of *hsp70-16* were significantly increased, whereas ratios of FAs in sepals of *vdac3* were significantly reduced (Fig. 2D; Table S3), again indicating that HSP70-16 and VDAC3 have different functions in the sepal’s HSR to mild HS.

### At 22 °C, *vdac3 *sepals experience more significant lipidomic changes than *hsp70-16* sepals

**Fig. 4:**
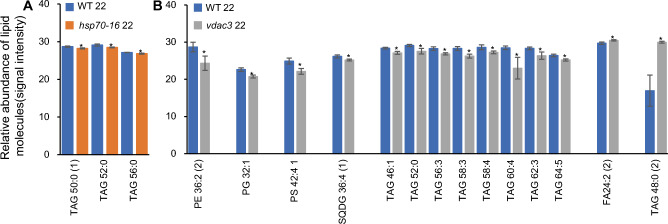
Effects of loss of function of *HSP70-16* or *VDAC3* on the levels of individual lipid molecules in sepals grown under 22 °C. **A** Significantly altered lipid molecules in sepals of *hsp70-16* as compared with wild-type (WT) sepals. **B** Significantly altered lipid molecules in sepals of *vdac3* as compared with wild-type (WT) sepals. * and **, significance at *P* < 0.05 and *P* < 0.001, respectively. Refer to legends in Fig. 1 for abbreviation name of each identified lipid class

Although loss-of-function of either *HSP70-16* or *VDAC3* did not significantly alter the total lipids in sepals grown under 22 °C (Fig. 2A), the effects of mutation in these two genes on individual lipid molecules remained unknown. Therefore, we looked closely at changes in identified lipid classes and lipid molecules and determined that *VDAC3* has stronger effects than *HSP70-16* on lipidomic profiles of sepals grown under 22 °C (Fig. 4; Table S4). We also confirmed the significant changes in the ratios of each lipid class, to total lipids, in *vdac3* sepals grown under 22 °C (Fig. 2C).

In *hsp70-16* sepals, although the ratio of each lipid class, to total lipids, was not significantly altered as compared with WT (Fig. 2C), levels (presented as signal intensities) of three saturated TAGs were slightly, but significantly, deceased as compared with WT (Fig. 4A; Table S4). In *vdac3* sepals, in contrast, except for MGDGs, ratios of all other detected ten lipid classes, to total lipids, were significantly reduced (Fig. 2C); correspondingly, levels of 14 lipid molecules were significantly altered, as compared with WT (Fig. 4B). Among them, levels of 12 lipids, including 7 unsaturated TAGs, were remarkably decreased, whereas those of one unsaturated long-chain FA (LCFA, FA24:2) and one saturated TAG (TAG 48:0) were significantly increased (Fig. 4B; Table S4). These results indicated that *VDAC3* is important in maintaining lipidomic compositions of sepals grown under normal temperatures.

### At 27 °C, *hsp70-16* sepals undergo much more extensive lipidomic changes than *vdac3* sepals

**Fig. 5:**
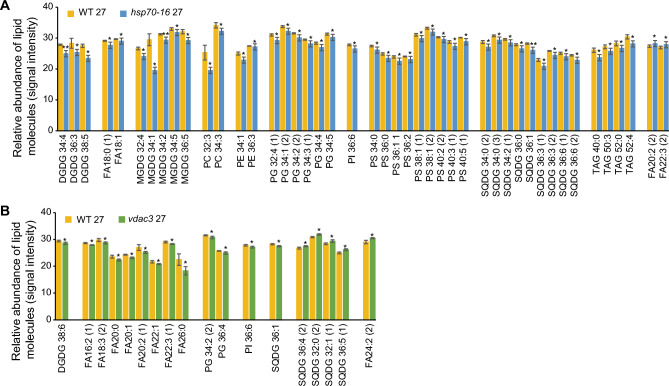
Effects of loss of function of *HSP70-16* or *VDAC3* on the levels of individual lipid molecules in sepals grown under 27 °C. **A** Significantly altered lipid molecules in sepals of *hsp70-16* as compared with wild-type (WT) sepals. **B** significantly altered lipid molecules in sepals of *vdac3* as compared with wild-type (WT) sepals. * and **, significance at *P* < 0.05 and *P* < 0.001, respectively. Refer to legends in Fig. 1 for abbreviation name of each identified lipid class

Different from the above-observed lipid changes in sepals grown at 22 °C, much more extensive lipidomic changes were detected in *hsp70-16* sepals, than in *vdac3* sepals, grown at 27 °C (Fig. 5; Table S4). In *hsp70-16* sepals, in addition to the above-mentioned significant decreases in levels of total lipids (Fig. 3), DGDGs, MGDGs, PCs, PSs, and SQDGs (Fig. 2B), and significantly increased ratios of FAs and TAGs to total lipids (Fig. 2D), levels of 45 lipid molecules (43 decreased and two increased, respectively) were significantly changed, as compared with WT (Fig. 5A; Table S4). These lipids covered all 11 identified lipid classes, except the DAGs, composed of mainly PLs, followed by GLs, SLs, and TAGs. Although levels of all other mentioned lipids were significantly reduced, those of two unsaturated FAs (FA 20:2 and FA 22:3) were significantly increased.

In *vadc3* sepals, levels of only 18 lipid molecules (13 decreased and five increased, respectively) were substantially altered as compared with WT (Fig. 5B; Table S4). Half of them were FAs, and levels of all these FAs, except FA 24:2, were significantly reduced. Remarkably, levels of four SLs were significantly increased in *vdac3* sepals. These results indicated that *HSP70-16* and *VDAC3* differently affect the sepal lipidomic response to mild HS temperature, with *HSP70-16* being more effective.

### Mild HS induces distinct lipidomic changes in sepals of WT, *hsp70-16,* and *vdac3*

**Fig. 6:**
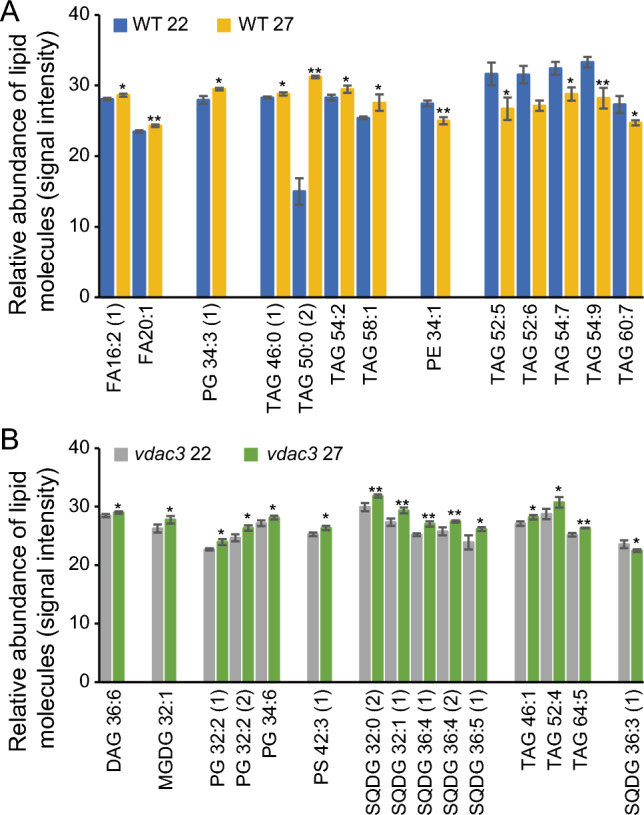
Effects of loss of function of *VDAC3* on the levels of individual lipid molecules in sepals grown under 27 °C as compared with those grown under 22 °C. A, significantly altered lipid molecules in sepals of WT plants grown under 27 °C as compared with those grown under 22 °C. B, significantly altered lipid molecules in sepals of *vdac3* plants grown under 27 °C as compared with those grown under 22 °C. * and **, significance at *P* < 0.05 and *P* < 0.001, respectively. Refer to legends in Fig. 1 for abbreviation name of each identified lipid class

In WT sepals grown under mild HS temperature, although total lipids and ratios of lipid classes to total lipids were not significantly changed, as compared with those in plants grown under normal temperature (Figs. 2, 3), levels of individual lipid molecules were dramatically altered (Fig. 6A; Table S5). These significantly altered lipid molecules, in WT sepals, included 13 lipid species, mainly TAGs, indicating a general role of TAGs in the sepal responses to HSR. Among these altered TAGs, levels of those with high unsaturated FAs were decreased, whereas those with saturated or low unsaturated FAs were increased, implying that WT sepals reduce unsaturation of lipids in response to mild HS.

In *vdac3* sepals grown under mild HS temperature, levels of 15 lipid molecules were significantly changed, with 14 being significantly increased and one significantly decreased, as compared with those grown under normal temperature (Fig. 6B; Table S5); notably, nine plastidic lipids (one MGDG, three PGs, and five SQDGs) were among these increased lipids. These results indicated that *vdac3* sepals accumulate plastidic lipids in response to mild HS.

**Fig. 7:**
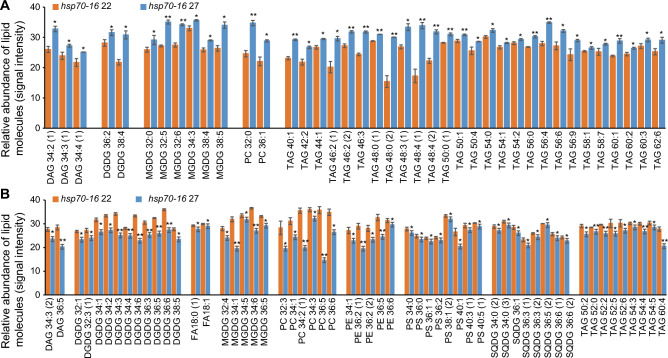
Effects of loss of function of *HSP70-16* on the levels of individual lipid molecules in sepals grown under 27 °C as compared with those grown under 22 °C. **A** Significantly increased lipid molecules in sepals of *hsp70-16* plants grown under 27 °C as compared with those grown under 22 °C. **B** Significantly decreased lipid molecules in sepals of *hsp70-16* plants grown under 27 °C as compared with those grown under 22 °C. * and **, significance at *P* < 0.05 and *P* < 0.001, respectively. Refer to legends in Fig. 1 for abbreviation name of each identified lipid class

In *hsp70-16* sepals grown under mild HS temperature, levels of 96 lipid molecules were significantly changed (40 were increased and 56 were decreased), as compared with those grown under normal temperature (Fig. 7; Table S5). Among them, levels of most TAGs were significantly increased, whereas those of FAs, PEs, PSs, and SQDGs were remarkably decreased. These results suggested a possible channeling of other lipids, such as FAs and PLs, to TAGs in *hsp70-16* sepals in response to mild HS.

## Discussion

Sepals are the outmost floral organs in Arabidopsis, with a unique cellular structure and chemistry, which play important roles in reproductive organ–environment interaction. In a previous study, we showed that *hsp70-16* mutants exhibit impaired epidermal cell structure and chemistry, in sepals, along with defective flower opening under both normal and mild HS growth temperatures; further, we determined that *HSP70-16* coordinately regulates flower development in response to both developmental and environmental cues (Chen et al. 2019; Ran et al. 2020).

The most significantly altered sepal chemistry, in *hsp70-16* sepals, is lipids, cell wall components, hormones, and ROS (Chen et al. 2019; Ran et al. 2020). In another study, we established that HSP70-16 interacts with VDAC3, jointly regulating ABA transport from the endosperm into the embryo and, thus, seed germination at 4 °C (Ashraf et al. 2021). These results established important roles played by *HSP70-16* and *VDAC3* in sepal responses to changing temperatures. Nevertheless, to what extent *HSP70-16* and its interactor *VDAC3* can affect the sepal lipid metabolism and HSR to mild HS temperature remained to be determined. To answer this question, in this study, we performed lipidomic profiling in sepals of WT, *hsp70-16*, and *vdac3* mutants, grown under both normal (22 °C) and mild HS (27 °C) temperatures, and revealed that *HSP70-16* and *VDAC3* distinctly affect the sepal lipidome and HSR to mild HS.

### The sepal lipidome is rich in LCFAs, VLCFAs, and SQDGs

Utilizing the power of UPLC–FT–MS, for the first time, we revealed the lipidome of *Arabidopsis* sepals, which constituted ten classes of glycerolipids and one class of FAs, the building block of glycerolipids. As two membrane lipids, sphingolipids and sterols, were absent from the internal library, we did not include data on sphingolipids and sterols, although both also play important roles in the plant stress response (Mlayeh et al. 2010; Reszczynska and Hanaka 2020; Sun et al. 2022). For the same reason, our lipidomic data did not have phospholipic acid (PA), important PLs in reproductive organs of *Arabidopsis thaliana* (Yunus et al. 2015).

The highest composition of detected sepal glycerolipids was the TAGs (about 25%), followed by PGs, SQDGs, FAs, DGDGs, MGDGs, PSs, PCs, PEs, and DAGs, whereas PIs were the lowest composition. Among them, plastidic lipids (DGDGs, MGDGs, SQDGs, and PGs) and PLs accounted for 31% and 21% of all detected lipids, respectively (Fig. 2C, D). Besides these common GLs and PLs, notably, more than 10% of the sepal lipidome was FAs, with different saturations (Table S1), covering from LCFAs (C16–C20) to very-long-chain FAs (VLCFAs, > C20) (Horn and Benning 2016). This observed sepal lipidomic profile differed from that reported in the *Arabidopsis* leaf (Shiva et al. 2020), flower (Nakamura et al. 2014), and seedling (Mueller et al. 2015), and also from that in tobacco pollen tubes (Krawczyk et al. 2022) and *Begonia grandis* leaves (Sun et al. 2022). Considering that lipid compositions and FA unsaturation levels determine membrane fluidity and stability (Upchurch 2008), and that both LCFAs and VLCFAs in membrane lipids are essential for membrane homeostasis (Batsale et al. 2021), the very sensitive nature of sepals to mild HS observed in our previous studies (Chen et al. 2019; Ran et al. 2020) could be explained, at least in part, by its unique lipidome, which is enriched with LCFAs and VLCFAs. In addition, the high ratio of SQDGs in the lipidome *Arabidopsis* sepals, likely due to the presence of chloroplasts in sepals, could also contribute to the very sensitive nature of sepals to mild HS, because SQDGs are indispensable for PSII activity in *Chlamydomonas reinhardtii* (Sato et al. 2003).

### WT sepals respond to mild HS by decreasing unsaturated TAGs

Although 27 °C is generally not regarded as a deleterious temperature for most plants, including *Arabidopsis* (Groot et al. 2017; Mittler et al. 2012; Sato et al. 2000), we observed that it significantly affects transcriptomes of WT sepals, indicating that 27 °C is a real HS temperature for sepals (Ran et al. 2020). As a consequence, under 27 °C, sepals in some flowers exhibit unbalanced lipid metabolism, leading to defective flower opening, due mainly to impaired cuticle patterning (Chen et al. 2019). In this study, our lipidomic results confirmed the deleterious effects of 27 °C on sepal chemistry, particularly lipid metabolism. As compared with WT sepals at 22 °C, WT sepals at 27 °C contained less unsaturated TAGs with two or three unsaturated bonds but more FAs with unsaturated bonds (Fig. 6A). This result corresponded well with the general notion that growth temperature modulates membrane lipid compositions, in plants, and that the degree of membrane lipid unsaturation decreases, in response to elevated temperatures (Falcone et al. 2004; Upchurch 2008). Our observed increase in unsaturated FAs in 27 °C sepals was consistent with the findings in moss (Legeret et al. 2016; Saidi et al. 2010). This result indicates the occurrence of a possible degradation of unsaturated complex lipids (such as TAGs) in mild HS stressed WT sepals.

An increase in saturated TAGs is also one of the adaptative characteristics of membrane lipids under HS (Falcone et al. 2004; Higashi et al. 2018; Krawczyk et al. 2022; Lu et al. 2020; Mueller et al. 2015; Sun et al. 2022). However, the heat-induced alterations of TAGs, and other glycerolipids, is generally not regarded as part of the genetically programmed HSR (Mueller et al. 2015), as they do not result from massive de novo FA synthesis, but rather from lipid remodeling (Krawczyk et al. 2022; Mueller et al. 2015).

Our previous transcriptomic study also showed that, as compared with WT sepals grown at 22 °C, although 11 lipid metabolic genes are significantly upregulated in WT sepals grown at 27 °C, none were involved in de novo biosynthesis of FAs and TAGs (Table S2 in Ran et al. 2020). Among them, six genes encoded lipid transport proteins, whereas four encoded phospholipases, including At1g76980 (patatin-like phospholipase domain protein), At4g38560 (phospholipase-like protein), DAD1-like lipase 1 (DALL1), and patatin-like protein 7 (PLP7), indicating an activated membrane lipid degradation and lipid transportation across membrane in WT sepals, upon mild HS. A significant upregulation of *PHYTYL ESTER SYNTHASE2* (*PES2*) expression in WT sepals, grown at 27 °C, also implied the accumulation of TAGs from DAGs that degraded from MGDGs, as reported in a previous study (Lippold et al. 2012). Therefore, our previous transcriptomic data and present lipidomic data, taken together, indicate a per se lipid remodeling in mild heat stressed WT sepals. Furthermore, the significant increment in saturated TAGs (particularly TAG 52:0 and TAG 46:0), together with a significant reduction in unsaturated TAGs (particularly TAG 54:7 and TAG 54:9), observed in 27 °C sepals (Fig. 6A), was  in line with findings in heat stressed *Chlamydomonas reinhardtii under* cells (Legeret et al. 2016), where HS-induced membrane lipid remodeling leads to accumulation of saturated TAGs.

Notably, although transcriptomic data indicated significant alterations in expression levels of genes involved in glycerolipid hydrolyzation, such as *At1g76980*, *At4g38560*, *DALL1*, and *PLP7* (Table S2 in Ran et al. 2020), significant lipidomic changes were not detected in the most abundant glycerolipid species, including PCs, MGDGs, DGDGs, PEs, and PGs in WT sepals under mild HS temperature (Figs. 2 and 6). These findings indicate that a mild HS does not lead to a dramatic disturbance in membrane lipid homeostasis, in WT sepals. This could be the consequence of the observed high level of PSs and SQDGs in sepals (Fig. 2; Table S1), as compared with the other tissues, such as leaves, roots, and flowers (Kehelpannala et al. 2021). Earlier, it is reported that HS induces increases in PSs, in leaves of many plant species with unknown function in HSR (Higashi and Saito 2019), and that SQDGs are involved in the structural integrity and heat tolerance of photosystem II in *Chlamydomonas reinhardtii* (Sato et al. 2003).

### *hsp70-16* sepals respond to mild HS by dramatically accumulating TAGs from both lipid remodeling and de novo biosynthesis

In contrast to WT, tremendous changes, not only in total lipids (Fig. 3), levels of five lipid glasses (Fig. 2B), and ratios of two lipid glasses to total lipids (Fig. 2D), but also levels of 96 lipid molecules (Fig. 7) were identified in *hsp70-16* sepals grown at 27 °C. In this scenario, a significant increment in TAGs was accompanied with remarkable reductions in plastidic lipids (MGDGs, DGDGs, and SQDGs except PG), extraplastidic lipids (PCs, Pes, and PSs), Fas (C18:0 and C18:1 FA), particularly eight Pes and eight SQDGs.

These findings indicate that *HSP70-16* is essential for the sepal responses to mild HS, and that loss-of-function of *HSP70-16* significantly alters lipidomic profiles, channeling plastidic lipids, and PLs to TAGs. This might well explain the imbalanced lipid metabolism, particularly disrupted cuticular lipid patterning (Ran et al. 2020), and more impeded flower opening in *hsp70-16* plants, when grown at 27 °C (Chen et al. 2019). Remarkably, as compared with *hsp70-16* sepals grown at 22 °C, expression levels of these 11 upregulated genes, responsible for membrane lipid degradation and lipid transportation in WT sepals grown at 27 °C, were not significantly changed in *hsp70-16* sepals grown at 27 °C; rather, the expression of *PLP2*, a homologue of *PLP7*, was significantly downregulated (Table S2 in Ran et al. 2020). These previous transcriptomic results indicate that an alternative pathway likely occurs to channel lipid metabolism to TAGs in *hsp70-16* sepals in response to mild HS. This notion gains support from the significant upregulation of expression of two TAG biosynthetic genes, *Δ9 stearoyl-ACP desaturase6* (*SAD6*) (Cai et al. 2020) and wax ester *synthase/diacylglycerol acyltransferase7* (*WSD7*) (Patwari et al. 2019) in *hsp70-16* sepals grown at 27 °C, as compared with those grown at 22 °C, in transcriptomic analyses (Table S2 in Ran et al. 2020). *SAD6* and *WSD7* participate in the biosynthesis of desaturated FAs and cuticular lipids, respectively, and both are reported to be involved in de novo biosynthesis of TAGs in plants (Cai et al. 2020; Patwari et al. 2019). Considering that biosynthesis of cuticular lipids and glycerolipids share C16–C18 FAs (Li-Beisson et al. 2010), and that loss-of-function of *HSP70-16* significantly altered expression levels of numerous cuticular lipid genes, thus changing pools of C16–C18 FAs, it is reasonable that accumulation of TAGs, in 27 °C *hsp70-16* sepals, is also likely from de novo biosynthesis.

Alternatively, as *HSP70-16*, as a chaperon, functions in the recovery of structures and functions of mild HS damaged proteins, including those for degradation of GLs and PLs and those for biosynthesis of FAs and TAGs, at this stage, the contributions of posttranscriptional regulations of those enzymes cannot be discounted. Post-transcriptional and/or post-translational regulation of lipidomic remodeling, in response to HS, has been reported in tobacco pollen tubes (Krawczyk et al. 2022). To comprehensively understand the role of *HSP70-16* in lipid remodeling and HSR to mild HS, biochemical characterization of the interaction between HSP70-16 and various proteins or enzymes, involved in biosynthesis of diverse lipids, is needed.

### *vdac3* sepals respond to mild HS by accumulating plastidial lipids

Since VDAC3 is an interactor protein of HSP70-16, and loss-of-function of *vdac3* promotes seed germination, under 4 °C (Ashraf et al. 2021), we included it in this study to ascertain whether VDAC3 affects the sepal lipidomic responses to mild HS, similar to those of HSP70-16. To our surprise, its effects on sepal responses to mild HS seemed to be different from those of HSP70-16. In response to mild HS, *vadc3* sepals accumulated three PGs and five SQDGs (Fig. 6). The increased PGs in *vdac3* sepals, in response to mild HS, are likely contributed by lipases (Higashi and Saito 2019) and PGs can modulate the plant response to temperature changes, by direct binding to important proteins, such as FLOWERING LOCUS T (FT) (Susila et al. 2021).

Compared with *HSP70-16* (Fig. 7), changes in the sepals lipidomic responses to mild HS, mediated by *VDAC3* (Fig. 6B), were not only quite limited, but were also rather distinct. Although accumulation of TAGs, MGDGs, and DAGs was observed in both *hsp70-16* and *vdac3* sepals, the increment of PCs and DGDGs was observed only in *hsp70-16* sepals, and increases of SQDGs and PGs were only seen in *vdac3* sepals (Figs. 6B, 7). In addition, compared with WT, loss-of-function of *VDAC3* seemed to have an affect mainly on SQDGs, MGDGs, and DAGs, in response to mild HS (Fig. 6A). These findings indicate a differential effect of *VDAC3* and *HSP70-16* on sepal lipidomic changes, in response to mild HS. Therefore, although *vdac3* showed a similar phenotype as that of *hsp70-16*, under 27 °C (Figure S1), physiological effects of the interaction of these two proteins on flower development under 27 °C remain unknown.

It is well known that membrane-localized VDCA proteins interact with various molecules, including lipids, to regulate a multitude of cellular functions, and that, conversely, membrane lipids, particularly PLs, control conductance, oligomerization, folding, channel-forming activity, voltage dependency, and the selectivity of VDAC proteins (Kanwar et al. 2020). The increase of SQDGs and PGs, in *vadc3* sepals at 27 °C, likely indicates a promoted prokaryotic (plastidial) pathway, by mild HS, whereas the decrease of SQDGs, in *hsp70-16* sepals at 27 °C, indicates an inhibited prokaryotic pathway, by mild HS (Reszczynska and Hanaka 2020), respectively. Biosynthesis of SQDGs is successively regulated by *UDP-Glc pyrophosphorylase 3* (*UGP3*), *UDP sulfoquinovose synthase1* (*SQD1*), and *SQD2* (Reszczynska and Hanaka 2020), and therefore, it is of interest to examine how *HSP70-16* and *VDAC3* differently affect the accumulation of SQDGs at the transcriptional level.

In summary, in response to mild HS, WT sepals change the degree of unsaturation of mainly TAGs, and *vadc3* sepals alter the degree of unsaturation of TAGs and SQDGs, whereas *hsp70-16* sepals remodel their lipidome and shift the de novo synthesis of TAGs and FAs from the chloroplast-based prokaryotic pathway to the endoplasmic reticulum-based eukaryotic pathway. Our study provides novel insights into the functions of *HSP70-16* and *VDAC3*, in response to mild HS, from a lipidomic prospective, which could facilitate a better understanding of lipids in plant development, in general, and in flower development, in particularly. Our findings also indicates that the sepal is a useful system for such studies.

## Experimental procedures

### Plant materials and growth conditions

All plants including wild-type (WT) Col-0 and two mutant plants of *hsp70-16* (SALK_028829C) and *vadc3* (SALK_127899C) were originally grown at 22 °C before bolting under a growth condition with a 16 h light/8 h night and 60% humidity. For mild HS temperature treatment, half of plants were kept in 22 °C, while another half of plants were transferred to and kept to grow at 27 °C in a growth incubator till the end of plant development with the same regime of the light and humidity as did as previously (Chen et al. 2019; Ran et al. 2020).

### Sample collection

Sepals from stage 12 buds of WT, *hsp70-16*, and *vdac3* plants grown under both 22 °C and 27 °C were harvested, freshly frozen with liquid nitrogen, and kept at − 80 °C until use. The flower developmental stage was defined as previously described (Smyth et al. 1990). For individual genotype, whether grown at 22 °C or 27 °C, four biological replicates were prepared. For individual replicate, 200–300 mg (fresh weight) of sepals were harvested from buds of different individual plants.

### Lipid extraction

Lipids were extracted from frozen sepal samples as previously described (Hummel et al. 2011; Hong et al. 2023). Briefly, sepal samples were milled under liquid nitrogen into fine powder, and 50 mg of frozen powder was weighted and homogenized using a ball mill (MM 301, Retsch, Düsseldorf, Germany) at maximum speed for twice (each 1 min). Lipids were then extracted with 1 mL of pre-cooled (− 20 °C) extraction buffer containing methanol/methyl tert-butyl ether (v/v, 1:3), in which 0.1 μg mL^−1^ of PE 34:0 and 0.1 μg mL^−1^ PC 34:0 (Sigma-Aldrich) were added as internal standards. Samples were incubated with extraction buffer at 4 °C for 10 min, followed by a sonication at room temperature for 10 min. 500 μL of 25% (v/v) aqueous methanol was then added, and samples were vortexed and centrifuged (14,000*g*) at 4 °C for 5 min. Finally, 500 μL of the upper phase was collected, dried under vacuum, and stored at − 80 °C until analysis.

### Lipidomic profiling

Lipidomic profiling was carried out using ultra-performance liquid chromatography–Fourier transform–mass spectrometry (UPLC–FT–MS, Waters) as described previously (Hummel et al. 2011). Briefly, lipophilic extracts were resuspended in 500 μL of buffer B (see below), and 1 μL of each was used for injection. The mobile phases included buffer A containing 1% 1 M ammonium acetate and 0.1% acetic acid in water and buffer B containing acetonitrile/isopropanol (v/v, 7:3) supplemented with 1 M ammonium acetate and 0.1% acetic acid. The gradient separation, carried out at a flow rate of 400 μL min^−1^, included an initial 1 min 45% A, 3 min linear gradient (from 45% A to 35% A), 8 min linear gradient (from 25 to 11% A), and 3 min linear gradient (from 11 to 1% A). After being cleaned up for 3 min with 1% A, the column was re-equilibrated for 4 min with 45% A.

Mass spectra were acquired with an Exactive mass spectrometer (Thermo Fisher, Waltham, USA) equipped with an electrospray ionization (ESI) interface in both positive and negative modes altering between full scan and all ion fragmentation scan mode (mass range 100–1500 m/z, capillary voltage 3.0 kV, sheath gas flow value 60, and auxiliary gas flow value 35), from minute 1 to minute 20 of the UPLC gradients. The resolution was 10,000 (10 scans s^−1^) and the Orbitrap loading time was up to 100 ms with a target value of 10^6^ ions. The capillary temperature and the temperature of the drying gas in the heated electrospray source were set to 150 °C and 350 °C, respectively, while the voltage of skimmer and the tube lens was set to 25 V and 130 V, respectively.

### Peak extraction, alignment, and annotation

Chromatogram processing, peak detection, and integration were performed automatically with REFINER MS 10.0 (GeneData, http://www.genedata.com) or manually with Xcalibur (Version 3.1, Thermo Fisher, Bremen, Germany), respectively, as described previously (Lapidot-Cohen et al. 2020). For further annotation, resulting features (m/z at a certain retention time) were queried against an in-house lipid library containing approximately 200 lipid species. For further validation of representatives of different lipid classes, MS/MS fragmentation using collision-induced dissociation mass spectra (25 eV collision energy) was used.

### Data normalization and statistical analysis

The peak intensity of each sample was normalized based on calculated chromatogram median intensity as described (Lapidot-Cohen et al. 2020). Excel (Microsoft^®^ Excel^®^ 2019MSO) was used for to calculate average and standard deviation, and to determine significant differences between treatments with a significance level of 0.05 (*P* < 0.05).

### Supplementary Information

Below is the link to the electronic supplementary material.

Supplementary file1 (PPTX 2845 KB)

Supplementary file2 (XLSX 55 KB)

## Data Availability

All relevant data are within the manuscript and its Supplementary files.
