# Supplementary material for: Lipidomic insights into the response of Arabidopsis sepals to mild heat stress
Source: aBIOTECH. 2023 Jun 6;4(3):224–37. doi: 10.1007/s42994-023-00103-x (PMC10638258; doi:10.1007/s42994-023-00103-x)
Supplement: Supplementary file 1 — Supplementary file1 (PPTX 2845 KB) [file 42994_2023_103_MOESM1_ESM.pptx]

## Slide 1
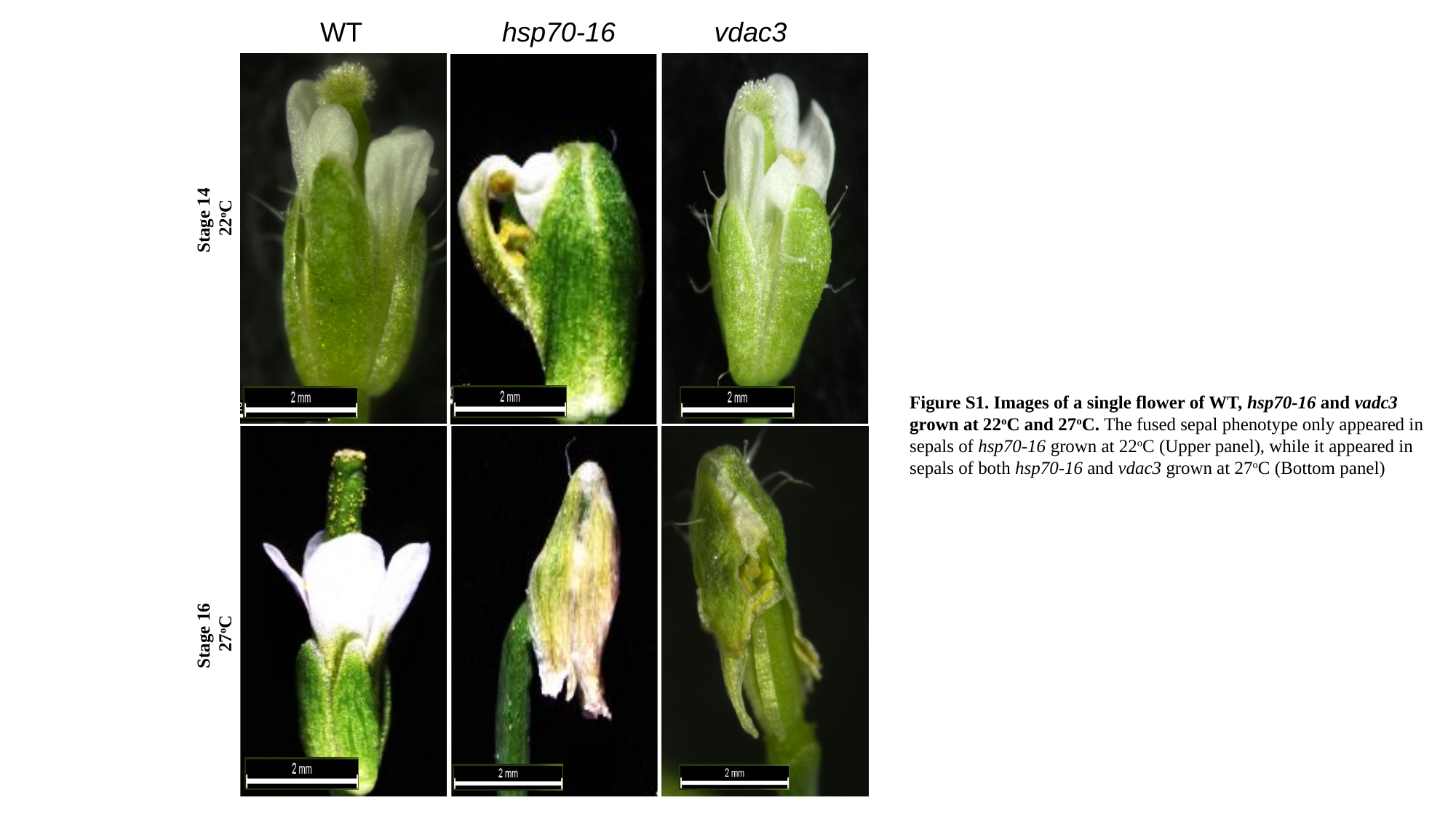

WT
hsp70-16
vdac3
Stage 14
 22oC
Figure S1. Images of a single flower of WT, hsp70-16 and vadc3 grown at 22oC and 27oC. The fused sepal phenotype only appeared in sepals of hsp70-16 grown at 22oC (Upper panel), while it appeared in sepals of both hsp70-16 and vdac3 grown at 27oC (Bottom panel)
Stage 16
27oC
